# Assessment of Immigrants’ Premium and Tax Payments for Health Care and the Costs of Their Care

**DOI:** 10.1001/jamanetworkopen.2022.41166

**Published:** 2022-11-09

**Authors:** Mark J. Ommerborn, Lynsie R. Ranker, Sharon Touw, David U. Himmelstein, Jessica Himmelstein, Steffie Woolhandler

**Affiliations:** 1 Institute for Community Health, Malden, Massachusetts; 2 Department of Community Health Sciences, Boston University School of Public Health, Boston, Massachusetts; 3 School of Urban Public Health, City University of New York at Hunter College, New York; 4 Department of Medicine, Cambridge Health Alliance, Cambridge, Massachusetts; 5 Department of Medicine, Harvard Medical School, Boston, Massachusetts

## Abstract

**Importance:**

Some worry that immigrants burden the US economy and particularly the health care system. However, no analyses to date have assessed whether immigrants’ payments for premiums and taxes that fund health care programs exceed third-party payers’ expenditures on their behalf.

**Objective:**

To assess immigrants’ net financial contributions to US health care programs.

**Design, Setting, and Participants:**

This cross-sectional analysis used 2017 data from the Medical Expenditure Panel Survey (MEPS) and the Current Population Survey (CPS) and 2014 to 2018 data from the American Community Survey. The main analyses used data from the calendar year 2017. Data from the calendar years 2012 to 2016 were also reported. Data were analyzed from June 15, 2020, to August 14, 2022. Participants comprised 210 669 community-dwelling respondents to the MEPS and CPS (main analysis) and nursing home residents who were included in the American Community Survey (additional analysis).

**Exposures:**

Citizenship and immigration status.

**Main Outcomes and Measures:**

Total and per capita payments for premiums and taxes that fund health care as well as third-party payers’ expenditures for health care in 2018 US dollars.

**Results:**

Among 210 669 participants, 51.0% were female, 18.3% were Hispanic, 12.3% were non-Hispanic Black, 60.3% were non-Hispanic White, and 9.2% were of other races and/or ethnicities. A total of 180 084 participants were respondents to the 2018 CPS, and 30 585 were respondents to the 2017 MEPS. Among the 180 084 CPS respondents, immigrants accounted for 14.1% (weighted to be nationally representative), with the subgroup of citizen immigrants accounting for 6.8%, documented noncitizen immigrants accounting for 3.7%, and undocumented immigrants accounting for 3.6%; US-born citizens constituted 85.9% of the population. Relative to US-born citizens, immigrants were more often age 18 to 64 years (79.6% vs 58.3%), of Hispanic ethnicity (45.0% vs 14.0%), and uninsured (16.8% vs 7.4%); similar percentages (51.4% vs 50.9%) were female. US-born citizens vs immigrants paid similar amounts in premiums and taxes ($6269 per capita [95% CI, $6185-$6353 per capita] vs $6345 per capita [95% CI, $6220-$6470 per capita]). However, third-party expenditures for immigrants’ health care ($5061 per capita; 95% CI, $4673-$5448 per capita) were lower than their expenditures for the care of US-born citizens ($6511 per capita; 95% CI, $6275-$6747 per capita). Immigrants, in general, paid significantly more per person (net contribution, $1284; 95% CI, $876-$1691) than was paid on their behalf. Most of this surplus was accounted for by undocumented immigrants, whose contributions exceeded their expenditures by $4418 per person (95% CI, $4047-$4789 per person). US-born citizens collectively paid $67.2 billion (95% CI, −$2.3 to $136.3 billion) less in premiums and taxes than third-party payers paid for their care. This deficit was mostly offset by the $58.3 billion (95% CI, $39.8-$76.8 billion) net surplus of payments from immigrants, 89% of which ($51.9 billion; 95% CI, $47.5-$56.3 billion) was attributable to undocumented immigrants.

**Conclusions and Relevance:**

In this study, immigrants appeared to subsidize the health care of other US residents, suggesting that concerns that immigrants deplete health care resources may be unfounded.

## Key Points

**Question:**

Do insurers and US government programs pay more for the care of immigrants than immigrants contribute to the health care system in insurance premiums and taxes?

**Findings:**

In this cross-sectional analysis of 210 669 respondents to the Medical Expenditure Panel Survey and the Current Population Survey, immigrants contributed $58.3 billion more in premiums and taxes in 2017 than insurers and government paid for their health care, and US-born citizens incurred a net deficit of $67.2 billion. Undocumented immigrants accounted for most (89.0%) of the surplus.

**Meaning:**

These findings suggest that immigrants, particularly undocumented immigrants, may subsidize the US health care financing system.

## Teaser

This cross-sectional study uses data from the Medical Expenditures Panel Survey and the Current Population Survey to assess immigrants’ net financial contributions to the US health care system.

## Introduction

Although immigrants use fewer health care services than US-born citizens,^1^ some worry that immigrants are a burden to the US economy and particularly to the health care system.^2^ Previous studies have found that immigrants,^3^ particularly undocumented immigrants,^4^ contribute more to the Medicare program (mostly through payroll taxes) than Medicare pays for their health care, thereby prolonging the life of the Medicare Trust Fund. Similarly, private health insurance premiums paid by immigrants (and by employers on their behalf) exceed the amounts private insurance plans pay for their care (ie, immigrants with private insurance effectively subsidize US-born private insurance enrollees).^5^ However, to our knowledge, no studies have examined the balance between immigrants’ financial contributions and costs to Medicaid, the third major pillar of the US health care economy, or provided an overall accounting of whether immigrants consume more health care than the care for which they pay.

This cross-sectional study assessed the net financial contributions of immigrants to US health care programs. Our analysis used person-level data on demographic characteristics, income, employment, and health care use along with detailed taxation models to calculate immigrants’ payments for premiums and taxes used to fund health care and the value of the care they received.

## Methods

### Study Data and Methods

The Cambridge Health Alliance Institutional Review Board does not consider analyses of deidentified public use data to be human participant research; thus, informed consent was not needed. The study followed the Strengthening the Reporting of Observational Studies in Epidemiology (STROBE) guideline for cross-sectional studies.

Participants comprised 210 669 community-dwelling respondents to the Medical Expenditure Panel Survey (MEPS) and Current Population Survey (CPS) (main analysis) and nursing home residents who were included in the American Community Survey (ACS) (additional analysis). We calculated total expenditures for the care of US-born citizens, all immigrants, and 3 immigrant subgroups: citizen immigrants, documented noncitizen immigrants, and undocumented immigrants. For each group and subgroup, we assessed their total financial contributions to all third-party payers (premium payments plus taxes) and their net contributions (defined as total contributions minus total expenditures by third-party payers on their behalf), which was our main outcome. Our main analyses used data from the calendar year 2017. We also reported data from the calendar years 2012 to 2016. Data were analyzed from June 15, 2020, to August 14, 2022.

### Main Data Sources

We used the 2017 MEPS^6^ to calculate health care expenditures for individuals and household-paid premiums, and we linked the MEPS to the 2016 National Health Interview Survey^7^ (NHIS; from which the MEPS sample is drawn), which provides information on nativity and citizenship. Using the 2018 Annual Social and Economic Supplement to the CPS,^8^ we assessed individuals’ incomes, states of residence, and their employers’ contributions to private insurance in 2017. In addition, we analyzed data from the 5-year 2014-2018 ACS^9^ to determine the immigration status of nursing home residents, whom the MEPS and CPS do not survey. We repeated all analyses for the years 2012 to 2016 (additional details on data sources and methods are provided in eMethods 1-5 in the Supplement).

### Immigration Status

For both the linked MEPS-NHIS data and the CPS data, we used questions on birthplace and citizenship to categorize individuals as US-born persons vs immigrants and citizen immigrants vs noncitizen immigrants. Using previously published methods^3,4,5^ adapted from Borjas,^10^ we then imputed the documentation status of noncitizen immigrants. In brief, we categorized all immigrants as documented or undocumented using place of birth; citizenship status; time in the US (in years); receipt of government benefits, such as Social Security, Medicare, or Medicaid; occupation requiring licensure; veteran status; employment by the federal government; and spouse’s citizenship or imputed documentation status. Our analysis departed from the Borjas algorithm^10^ by omitting Section 8 housing and rental subsidies and a birthplace of Cuba because these variables were not available in MEPS public use files.

### Expenditures for Care

Using the MEPS, we summed third-party (Medicare, Medicaid, and private insurer) expenditures for health care incurred by community-dwelling individuals according to immigration status. For nursing home residents, we used ACS data to assess the proportion of these residents accounted for by persons in each immigration status group and attributed total nursing home expenditures reported in the National Health Expenditure Accounts (NHEA) to each group according to that proportion.

Expenditures reported in the MEPS are lower than actual spending recorded in the NHEA because (1) MEPS expenditures exclude third-party payers’ overhead and (2) patients and services incurring high costs are known to be underrepresented in the MEPS.^11,12^ To account for this discrepancy, we adjusted expenditures upward to match the NHEA data.

### Premium Contributions

We calculated private insurance premium contributions for each immigration group by summing household-paid premiums reported in the MEPS and employer-paid premiums reported in the CPS, with adjustments for the top-coding of premiums (ie, the practice of calculating the upper limit of all published premium values and either replacing any premium value that is greater than the upper limit with the upper limit value or not publishing that premium value in the file or database) in the CPS and adjustments to match the private insurance revenues reported in the NHEA. Enrollees’ premiums for Medicare Part B and Part D coverage are income based; for enrollees in those programs, we estimated premiums using income data from the MEPS. For individuals reporting coverage by Affordable Care Act (ACA) exchange insurance in the MEPS, we estimated their personal contribution (ie, their payments excluding federal subsidies) based on their income relative to the federal poverty level and a published estimate of exchange subsidy amounts.^13^

### Tax Contributions to Health Care

Medicare Part A is supported by payroll taxes and, to a lesser extent, by taxes on the Social Security benefits of higher-income beneficiaries. We estimated individuals’ contributions to Medicare Part A by multiplying their wage or salary earnings (reported in the CPS) by 2.9% (the usual Medicare payroll tax rate). We then added an estimate of taxes on Social Security income based on tax rates from a Congressional Research Service analysis.^14^

General tax revenues are used to finance Medicare Part B, the federal government’s proportion of Medicaid, and subsidies for ACA marketplace plans. We determined federal spending for these programs using NHEA data and calculated individuals’ tax contributions to the programs by applying Congressional Budget Office data^15^ on federal general tax rates according to their income reported in the CPS.

The types of taxes that fund states’ shares of Medicaid and the extent of Medicaid spending vary by state. For each state, we determined the amount of state taxes allocated to Medicaid using data from the Kaiser Family Foundation on total Medicaid (including Emergency Medicaid programs, through which individual states provide limited coverage for emergency medical services to noncitizens who would qualify for full Medicaid benefits if not for their immigration status^16^) spending in each state,^17^ adjusted downward for the federal share of Medicaid based on each state’s federal Medicaid assistance percentage. We then used state-level tax rate data from the Institute on Taxation and Economic Policy^18^ and individual-level income data and state of residence from the CPS to estimate each individual’s state tax contributions to Medicaid.

Because the MEPS does not provide cost estimates for uncompensated care delivered at private facilities, we used an American Hospital Association estimate of uncompensated care expenditures ($38.4 billion, or 3.6% of total hospital expenditures^19^). We then distributed funding contributions among citizen and immigrant groups in proportion to their contributions to the other third-party payers combined, incorporating the assumption that other third-party payers would ultimately fund uncompensated care. We distributed uncompensated care expenditures among the groups according to each group’s proportion of the total uninsured population, which we calculated from the CPS data. For instance, undocumented immigrants accounted for 15.8% of the uninsured population in 2017; hence, undocumented immigrants were assigned 15.8% of all uncompensated care expenditures.

### Statistical Analysis

All values were adjusted for inflation to 2018 dollars using the Consumer Price Index.^20^ Analyses were performed using SAS software, version 9.4 (SAS Institute Inc), applying sampling weights that allowed national estimates and using statistical procedures appropriate for complex samples. The significance threshold was 2-tailed *P* = .05.

## Results

### Population Characteristics

Among 210 669 participants, 51.0% were female, 49.0% were male, 18.3% were Hispanic, 12.3% were non-Hispanic Black, 60.3% were non-Hispanic White, and 9.2% were of other races and/or ethnicities. A total of 180 084 participants were respondents to the 2018 CPS, and 30 585 were respondents to the 2017 MEPS. The (weighted) demographic characteristics of respondents to the 2018 CPS vs respondents to the 2017 MEPS were almost identical (Table 1). Among the 180 084 respondents to the CPS, immigrants accounted for 14.1% (weighted to be nationally representative) of the population, with the subgroup of citizen immigrants accounting for 6.8%, documented noncitizen immigrants accounting for 3.7%, and undocumented immigrants accounting for 3.6%; US-born citizens constituted 85.9% of the population (Table 2). Relative to US-born citizens, immigrants were more often age 18 to 64 years (79.6% vs 58.3%), of Hispanic ethnicity (45.0% vs 14.0%), and uninsured (16.8% vs 7.4%); similar percentages (51.4% vs 50.9%) were female.

**Table 1.** Demographic Characteristics by Nativity Status for Current Population Survey and Medical Expenditures Panel Survey Samples, 2017

Characteristic	Participants, %									
	CPS respondents (n = 180 084)					MEPS respondents (n = 30 585)				
	All immigrants (n = 24 729)	Citizen immigrants (n = 11 693)	Documented noncitizen immigrants (n = 6640)	Undocumented immigrants (n = 6396)	US-born citizens (n = 155 355)	All immigrants (n = 5745)	Citizen immigrants (n = 2711)	Documented noncitizen immigrants (n = 2519)	Undocumented immigrants (n = 515)	US-born citizens (n = 24 840)
Age group, y										
0-17	5.7	3.1	9.2	7.1	25.7	5.6	4.2	6.9	7.3	25.8
18-39	35.4	24.3	38.6	52.7	28.6	34.4	23.9	44.3	52.3	28.3
40-64	44.2	50.5	38.5	38.2	29.7	44.6	49.5	39.5	37.7	29.9
≥65	14.6	22.1	13.6	1.9	16.0	15.5	22.4	9.2	2.7	16.0
Sex										
Female	51.4	53.8	53.3	44.9	50.9	51.9	52.9	53.5	40.6	50.9
Male	48.6	46.2	46.7	55.1	49.1	48.1	47.1	46.5	59.4	49.1
Race and ethnicity										
Hispanic	45.0	34.6	52.5	56.8	14.0	45.8	37.6	57.4	45.7	13.6
Non-Hispanic Black	8.8	10.6	9.3	5.0	12.9	8.2	9.3	7.5	4.7	13.0
Non-Hispanic White	18.8	22.9	17.4	12.8	67.3	16.9	21.1	11.3	15.9	67.1
Other^a^	27.3	31.9	20.9	25.4	5.9	29.1	32.0	23.8	33.7	6.3
Primary health insurance										
Private	55.0	57.4	43.6	61.9	58.7	52.6	57.4	42.3	65.2	59.9
Medicare	14.6	22.3	15.0	0	17.5	16.5	24.8	9.3	0	18.8
Medicaid or other government plan	13.6	11.4	31.1	0.1	16.4	13.9	11.3	20.8	2.0	16.4
Uninsured	16.8	8.9	10.2	38.1	7.4	17.1	6.5	27.6	32.8	4.9
Time in US, y										
≤10	26.5	9.6	39.4	45.2	NA	23.3	8.4	35.5	53.3	NA
>10	73.5	90.4	60.6	54.8	NA	74.7	91.6	64.5	46.7	NA
Citizen	48.1	100	0	0	100	44.2	100	0	0	100

Abbreviations: CPS, Current Population Survey; MEPS, Medical Expenditure Panel Survey; NA, not applicable.

^a^ Other races and ethnicities include American Indian or Alaska Native, Asian or Pacific Islander, and multiple races and/or ethnicities.

**Table 2.** Proportion of Immigrants and US-Born Citizens in the US Population and Contributions to and Expenditures From Third-Party Payers, 2017

Group	US population		Contribution		Expenditure		Net contribution (95% CI), $ in billions^a^
	Individuals, No. (95% CI), millions	Individuals, %	Amount (95% CI), $ in billions	Amount, %	Amount (95% CI), $ in billions	Amount, %	
All immigrants	45.4 (44.8 to 46.0)	14.1	288.1 (282.4 to 293.8)	14.2	229.8 (212.2 to 247.4)	11.3	58.3 (39.8 to 76.8)
Citizen immigrants	21.9 (21.4 to 22.3)	6.8	165.9 (161.7 to 170.0)	8.2	161.5 (145.0 to 178.1)	7.9	4.3 (−12.8 to 21.4)
Documented noncitizen immigrants	11.8 (21.4 to 22.3)	3.7	57.7 (55.5 to 59.9)	2.8	55.6 (50.3 to 60.9)	2.7	2.1 (−3.7 to 7.8)
Undocumented immigrants	11.7 (11.4 to 12.1)	3.6	64.5 (61.6 to 67.4)	3.2	12.6 (9.4 to 15.9)	0.6	51.9 (47.5 to 56.3)
US-born citizens	277.7 (276.7 to 278.8)	85.9	1741.2 (1718.0 to 1764.4	85.8	1808.4 (1742.9 to 1874.0)	88.7	−67.2 (−136.8 to 2.3)

^a^ Calculated as contributions minus expenditures.

### Total Contributions, Expenditures, and Net Contributions

In 2017, immigrants’ and nonimmigrants’ proportions of contributions to premiums and taxes to fund health care were similar to their proportions of the population. In total, immigrants paid 14.2% ($288.1 billion; 95% CI, $282.4-$293.8 billion) of the total premiums and taxes for health care, whereas US-born citizens contributed 85.8% ($1741.2 billion; 95% CI, $1718.0-$1764.4 billion) of the total (Table 2). Among immigrant subgroups, citizen immigrants contributed 8.2% ($165.9 billion; 95% CI, $161.7-$170.0 billion) of the total, documented noncitizen immigrants contributed 2.8% ($57.7 billion; 95% CI, $55.5-$59.9 billion), and undocumented immigrants contributed 3.2% ($64.5 billion; 95% CI, $61.6-$67.4 billion).

Although the proportions of health care expenditures among US-born citizens (88.7%; $1808.4 billion; 95% CI, $1742.9-$1874.0 billion), citizen immigrants (7.9%; $161.5 billion; 95% CI, $145.0-$178.1 billion), and documented noncitizen immigrants’ (2.7%; $55.6 billion; 95% CI, $50.3-$60.9 billion) were approximately commensurate with their proportions of the population (US-born citizens: 85.9% [277.7 million individuals; 95% CI, 276.7-278.8 million individuals]; citizen immigrants: 6.8% [21.9 million individuals; 95% CI, 21.4-22.3 individuals; documented noncitizen immigrants: 3.7% [11.8 million individuals; 95% CI, 21.4-22.3 million individuals]), undocumented immigrants’ proportion of expenditures (0.6% of the total; $12.6 billion; 95% CI, $9.4-$15.9 billion) was one-sixth of their proportion of the population (3.6%; 11.7 million individuals; 95% CI, 11.4-12.1 million individuals).

US-born citizens collectively paid $67.2 billion (95% CI, −$2.3 to $136.8 billion) less in premiums and health care taxes than third-party payer expenditures for their care, although CIs were wide. This deficit was mostly offset by immigrants’ significant net surplus ($58.3 billion; 95% CI, $39.8-$76.8 billion), 89% of which ($51.9 billion; 95% CI, $47.5-$56.3 billion) was attributable to undocumented immigrants.

### Per Capita Contributions, Expenditures, and Net Contributions

Results from 2017 expressed on a per capita basis are shown in Table 3. Although immigrants vs US-born citizens paid similar amounts in premiums and taxes ($6345 per capita [95% CI, $6220-$6470 per capita] vs $6269 per capita [95% CI, $6185 to $6353 per capita]), the amount paid by third-party payers for immigrants’ care ($5061 per capita; 95% CI, $4673-$5448 per capita) was 22.3% lower than the costs incurred for the care of US-born citizens ($6511 per capita; 95% CI, $6275-$6747 per capita). Thus, immigrants, in general, contributed $1284 (95% CI, $876-$1691) more per person than was paid on their behalf. Most of this surplus was accounted for by undocumented immigrants, whose contributions exceeded their expenditures by $4418 per person (95% CI, $4047-$4789 per person); comparable figures were $175 per person (95% CI, −$312 to $663 per person) for documented noncitizen immigrants and $197 per person (95% CI, −$585 to $979 per person) for citizen immigrants. US-born citizens incurred a negative net contribution of $242 per person (95% CI, −$492 to $8 per person).

**Table 3.** Per Capita Contributions to and Expenditures From Third-Party Payers Among Immigrants and US-Born Citizens, 2017

Group	Per capita amount (95% CI), $		
	Contribution	Expenditure	Net contribution^a^
All immigrants	6345 (6220 to 6470)	5061 (4673 to 5448)	1284 (876 to 1691)
Citizen immigrants	7589 (7400 to 7779)	7392 (6633 to 8151)	197 (−585 to 979)
Documented noncitizen immigrants	4889 (4701 to 5077)	4713 (4263 to 5162)	175 (−312 to 663)
Undocumented immigrants	5493 (5246 to 5739)	1075 (798 to 1352)	4418 (4047 to 4789)
US-born citizens	6269 (6185 to 6353)	6511 (6275 to 6747)	−242 (−492 to 8)

^a^ Calculated as contributions minus expenditures.

### Contributions, Expenditures, and Net Contributions According to Payer

In 2017, payments for the care of US-born citizens by each major third-party payer (Medicare, Medicaid, and private insurers) exceeded those persons’ contributions to those payers (net contributions to Medicare: −$19.2 billion [95% CI, −$59.5 to $21.1 billion]; Medicaid: −$13.7 billion [95% CI, −$22.1 to −$5.2 billion]; private insurers: −$39.3 billion [95% CI, −$93.3 to $14.7 billion]) (Table 4). In contrast, immigrants had net surpluses for all of those payers (net contributions to Medicare: $13.7 billion [95% CI, −$1.3 to $28.7 billion]; Medicaid: $10.2 billion [95% CI, $6.9-$13.6 billion]; private insurers: $39.3 billion [95% CI, $29.1-$49.5 billion]), and a small net deficit in uncompensated care (−$4.9 billion; 95% CI, −$5.7 to −$4.1 billion).

**Table 4.** Contributions to and Expenditures From Third-Party Payers, by Payer, Among Immigrants and US-Born Citizens, 2017

Group	Amount (95% CI), $ in billions		
	Contribution	Expenditure	Net contribution^a^
**All immigrants**			
Medicare	98.0 (95.5 to 100.4)	84.2 (69.4 to 99.0)	13.7 (−1.3 to 28.7)
Private	100.6 (96.3 to 105.0)	61.4 (52.1 to 70.6)	39.3 (29.1 to 49.5)
Medicaid	84.1 (81.5 to 86.6)	73.8 (71.6 to 76.0)	10.2 (6.9 to 13.6)
Uncompensated care	5.5 (4.7 to 6.2)	10.4^b^	−4.9 (−5.7 to −4.1)^c^
**Citizen immigrants**			
Medicare	58.2 (56.2 to 60.1)	67.8 (53.7 to 82.0)	−9.6 (−23.9 to 4.6)
Private	58.7 (55.6 to 61.8)	43.3 (34.8 to 51.7)	15.4 (6.4 to 24.4)
Medicaid	45.8 (44.0 to 47.7)	47.8 (45.8 to 49.8)	−2.0 (−4.7 to 0.7)
Uncompensated care	3.1 (2.8 to 3.5)	2.6^b^	0.5 (0.2 to 0.8)^c^
**Documented noncitizen immigrants**			
Medicare	18.9 (18.0 to 19.8)	16.4 (11.8 to 20.9)	2.5 (−2.1 to 7.2)
Private	20.8 (19.1 to 22.6)	11.7 (9.1 to 14.2)	9.2 (6.1 to 12.3)
Medicaid	16.9 (15.9 to 17.9)	26.0 (25.0 to 26.9)	−9.1 (−10.4 to −7.7)
Uncompensated care	1.1 (1.0 to 1.2)	1.6^b^	−0.6 (−0.6 to −0.5)^c^
**Undocumented immigrants**			
Medicare	20.9 (19.8 to 21.9)	0.04 (0.0 to 0.1)	20.8 (19.7 to 21.9)
Private	21.1 (18.9 to 23.4)	6.4 (3.2 to 9.7)	14.7 (10.7 to 18.6)
Medicaid	21.3 (19.8 to 22.8)	0.1 (0.1 to 0.1)	21.3 (19.8 to 22.8)
Uncompensated care	1.2 (1.1 to 1.3)	6.1^b^	−4.9 (−5.0 to −4.8)^c^
**US-born citizens**			
Medicare	615.7 (609.2 to 622.3)	634.9 (595.2 to 674.6)	−19.2 (−59.5 to 21.1)
Private	599.9 (584.6 to 615.3)	639.2 (587.4 to 691.0)	−39.3 (−93.3 to 14.7)
Medicaid	492.6 (486.9 to 498.3)	506.3 (500.1 to 512.4)	−13.7 (−22.1 to −5.2)
Uncompensated care	32.9 (17.8 to 48.1)	28.0^b^	4.9 (−10.2 to 20.0)^c^

^a^ Calculated as contributions minus expenditures.

^b^ 95% CIs were not available because the American Hospital Association did not report CIs for its estimates of total uncompensated care (from which the point estimates were calculated for this study).

^c^ 95% CIs were slightly underestimated because they did not account for uncertainty in the uncompensated care expenditure estimates.

Undocumented immigrants incurred surpluses of contributions greater than the costs of their care for all payers (Medicare: $20.8 billion [95% CI, $19.7-$21.9 billion]; Medicaid: $21.3 billion [95% CI, $19.8-$22.8 billion], private insurers: $14.7 billion [95% CI, $10.7-$18.6 billion]) with the exception of uncompensated care (−$4.9 billion; 95% CI, −$5.0 to −$4.8 billion), whereas documented noncitizen immigrants incurred a deficit for Medicaid (−$9.1 billion; 95% I, −$10.4 to −$7.7 billion) and a surplus for private insurance ($9.2 billion; 95% CI, $6.1-$12.3 billion).

### Net Contributions to Health Care Financing, 2012-2017

Between 2012 and 2016, immigrants’ contributions to third-party payers exceeded expenditures on their behalf in each year ($18.1 billion in 2012, $23.0 billion in 2013, $17.7 billion in 2014, $18.8 billion in 2015, and $48.2 billion in 2016). As observed in our main (2017) analysis, undocumented immigrants accounted for most or all of these net contributions ($30.2 billion in 2012, $25.5 billion in 2013, $35.6 billion in 2014, $41.9 billion in 2015, and $50.1 billion in 2016).

Overall, from 2012 to 2017, immigrants paid $184.2 billion more in premiums and taxes than the costs incurred by third-party payers for their care. In contrast, during that time, US-born citizens incurred net contribution deficits totaling $185.2 billion (−$49.0 billion in 2012, −$19.0 billion in 2013, −$34.4 billion in 2014, −$11.6 billion in 2015, −$4.0 billion in 2016, and [as reported in a previous paragraph] −$67.2 billion in 2017).

## Discussion

This cross-sectional study found that immigrants contributed more in premiums and taxes than private insurers and public programs paid for their health care. Although immigrants and US-born citizens paid similar amounts (per capita) into the health care system, the total amount paid by third-party payers for immigrants’ care was 22.3% lower. Undocumented immigrants accounted for most of the net contribution surplus among all immigrants. In other words, immigrants, especially undocumented immigrants, subsidized the US health care financing system.

Several factors likely underlie immigrants’ lower use of health care services. Fewer immigrants are older adults or nursing home residents, and almost none are newborns. Recent immigrants are, in general, healthier than other US residents of similar age.^21^ Immigrants who do not speak English often encounter language and cultural barriers that deter health care use.^22^ In addition, laws restrict immigrants’ enrollment in tax-funded programs like Medicare, ACA-subsidized marketplace coverage, and Medicaid,^23^ although Emergency Medicaid programs often cover maternity and emergency care for noncitizen immigrants (expenditures that would likely have been counted in our analysis).

Immigrants’ substantial contributions to health care funding (despite their relatively low incomes) may be associated with their high labor force participation rate, particularly among men who have recently arrived in the US. Hence, they and their employers (whose benefit payments are widely considered part of the employee’s earned compensation) contribute to health insurance premiums as well as payroll and other taxes.

Although few studies have addressed immigrants’ net contributions to the health care sector,^3,4,5^ more studies have assessed their net fiscal contributions (defined as taxes paid minus government costs for publicly funded services used).^24,25^ The Organization for Economic Co-operation and Development concluded that, between 2006 and 2018, immigrants’ net fiscal contributions were positive in the US (and in most other wealthy nations), both overall and specifically for publicly funded health care.^25^ According to estimates from the Organization for Economic Co-operation and Development, immigrants’ contributions to government-funded health care programs in the US exceeded their benefit costs by 16%,^25^ which was similar to our estimate.

Our findings have implications not only for health care financing, but for debates on immigration policy in general. Trump administration officials cited “protecting taxpayers by ensuring people who are immigrating to this country don’t become public burdens”^26^ as the motivation for widening public charge sanctions to deter immigrants from enrolling in public nutrition and health care programs. A leading prime-time news personality has asserted that undocumented immigrants have “plundered” America, in part by taking advantage of generous government benefits like “free health care.”^27^ Our estimates suggest that these concerns may be unfounded; rather than depleting health care resources, immigrants appear to subsidize them.

### Limitations

This study has several limitations. The surveys we analyzed asked respondents about their birthplace, years of residence in the US, citizenship status, and multiple factors associated with documentation status, but they did not directly ask respondents about their documentation status. Although the method we used to impute documentation status has been widely used, this method and similar approaches are imprecise.^28^ Some recent studies^21,29^ have developed machine learning algorithms to impute the documentation status of adults (but not children), which may be more accurate. Although our methods likely misclassified some individuals (eg, undocumented immigrants who were aware of their short-term coverage by Emergency Medicaid may have been misclassified as documented noncitizen immigrants), our population-wide estimates of immigrant subgroups were similar to those of the Pew Research Center^30^ (Table in eMethods 1 in the Supplement).

Our estimates of contributions to Medicare and private insurance rely on straightforward calculations of tax and premium payments (although the CPS data on employer contributions to premiums are imputed by the US Census Bureau based on previous patterns of such contributions).^31^ However, our findings regarding contributions to Medicaid are less certain. We calculated individuals’ state and local tax contributions to health care programs for each state assuming uniformity within the state, although taxes paid to local governments vary. Similarly, we assumed a uniform federal Medicaid match rate for funding in each state, although some state and local Medicaid expenditures for immigrants are ineligible for matching funds. Our analysis accounts for federal but not state-funded subsidies for ACA exchange plans. Overall, errors in our estimates of tax contributions for state-funded programs are likely small because the components for which we lacked precise data accounted for a small proportion of health care funding.

Our estimates of nursing home expenditures incorporate the reasonable but unproven assumption that expenditures on behalf of immigrant residents are proportionate to their proportion of the nursing home population. In other words, we assumed that per-resident costs for nursing home care would be similar regardless of immigration status.

Some undocumented persons may have been unaware of coverage available through Emergency Medicaid programs (which accounted for approximately 0.3% of Medicaid expenditures^32^), producing a small underestimation of Medicaid expenditures and a small overestimation of uncompensated care expenditures for their health care. No data were available for medical expenditures on behalf of incarcerated immigrants. However, expenditures for prisoners’ care account for only 0.003% of total health care spending.^33^

No fully reliable data on uncompensated care are available.^34^ The American Hospital Association data we used, although similar to other estimates,^35^ did not (1) encompass care delivered by nonhospital practitioners, (2) separate uncompensated care from bad debt (much of which is attributable to insured patients who are unable to pay copayments and deductibles), or (3) allocate uncompensated care to specific groups. We conservatively assumed that, among uninsured persons, immigrants would use the same amount of uncompensated care as US-born citizens. If, as seems possible, uninsured immigrants are more reticent to seek care, that would result in overestimation of immigrants’ proportion of uncompensated health care expenditures.^36,37^

Our findings exclude most revenues and expenditures for military service members and individuals receiving care through the Department of Veterans Affairs or the Indian Health Service, likely producing underestimation of immigrants’ net contributions given that they contribute taxes to fund these programs, but relatively few qualify for benefits under them.^38^ In addition, we excluded the relatively modest spending for some other public programs. Because we assumed that out-of-pocket payments for care and the value of such care were equivalent and hence could not represent cross-subsidies between groups, we excluded them from our calculations. Including them would have increased our 2017 estimates of total expenditures and total contributions by 14.7% but would not have altered our estimates of net contributions. Our estimates of contributions to third-party payers and those payers’ expenditures for care are not expected to match, in part because some federal spending is funded by borrowing rather than current revenues.

Our estimates do not reflect policy changes since 2017 (eg, California’s expansion of Medicaid coverage to some undocumented immigrants^39^ or immigrants’ avoidance of care due to changes in the public charge rule).^26^ The CPS stopped reporting employers’ premium contributions after 2017, precluding analysis of data from more recent years.

## Conclusions

 This cross-sectional study found that immigrants contributed more in insurance premiums and taxes than private insurers and government programs paid on their behalf. These findings suggest that concerns about immigrants depleting health care resources may be unfounded and that immigrants instead appear to subsidize the US health care financing system.

## Notes

Supplement. **eMethods 1.** Imputation for Determining Immigration and Documentation Status **eMethods 2.** Methods Used to Estimate Private Insurance Contributions and Expenditures **eMethods 3.** Methods Used to Estimate Contributions to and Expenditures by Medicare **eMethods 4.** Methods Used to Estimate Contributions to and Expenditures by Medicaid **eMethods 5.** Methods Used to Estimate Uncompensated Care Expenditures
