# Supplementary material for: Assessment of Immigrants’ Premium and Tax Payments for Health Care and the Costs of Their Care
Source: JAMA Netw Open. 2022 Nov 9;5(11):e2241166. doi: 10.1001/jamanetworkopen.2022.41166 (PMC9647478; doi:10.1001/jamanetworkopen.2022.41166)
Supplement: Supplement. — eMethods 1. Imputation for Determining Immigration and Documentation Status eMethods 2. Methods Used to Estimate Private Insurance Contributions and Expenditures eMethods 3. Methods Used to Estimate Contributions to and Expenditures by Medicare eMethods 4. Methods Used to Estimate Contributions to and Expenditures by Medicaid eMethods 5. Methods Used to Estimate Uncompensated Care Expenditures [file jamanetwopen-e2241166-s001.pdf]

Supplemental Online Content

Ommerborn MJ, Ranker LR, Touw S, Himmelstein DU, Himmelstein J, Woolhandler S. Assessment of immigrants' premium and tax payments for health care and the costs of their care. *JAMA Netw Open*. 2022;5(11):e2241166. doi:10.1001/jamanetworkopen.2022.41166

eMethods 1. Imputation for Determining Immigration and Documentation Status

eMethods 2. Methods Used to Estimate Private Insurance Contributions and Expenditures

eMethods 3. Methods Used to Estimate Contributions to and Expenditures by Medicare

eMethods 4. Methods Used to Estimate Contributions to and Expenditures by Medicaid

eMethods 5. Methods Used to Estimate Uncompensated Care Expenditures

This supplemental material has been provided by the authors to give readers additional information about their work.

© 2022 Ommerborn MJ et al. *JAMA Network Open*.

eMethods 1. Imputation for Determining Immigration and Documentation Status

The Current Population Survey (CPS) includes information on citizenship and nativity status for all respondents. To ascertain citizenship and nativity status for Medical Expenditure Panel Survey (MEPS) respondents we merged the MEPS record with their National Health Interview Survey record (from which the MEPS sample is drawn) which included information on nativity and citizenship status. We considered all persons born outside of the United States (US) to be immigrants.

We used previously published methods[1-3] adopted from Borjas[4] to determine the documentation status of non-citizen immigrants. The method classifies immigrants' documentation status using information on citizenship status, age, place of birth, length of time in the US, occupation requiring government licensure, spouse's citizenship or documentation status, and receipt of public benefits such as social security, Medicare, or Medicaid. The Borjas calculation uses additional information on Section 8 housing and rental subsidies and data on birthplace of Cuba, which are not available in the MEPS or CPS. Hence, we modified our procedures to exclude these indicators.[5] Our estimates of undocumented immigrants yield estimates similar to those published by the Pew Research Center (eTable).[6]

eTable. Comparison of the undocumented immigrant population calculation in our methods to the Pew Research Center, in millions

	Our method	Pew Research Center*
2012	12.3	11.2
2013	11.8	11.2
2014	11.5	11.1
2015	11.6	11.0
2016	11.9	10.7
2017	11.7	10.5

* https://www.pewresearch.org/hispanic/interactives/unauthorized-trends/

© 2022 Ommerborn MJ et al. *JAMA Network Open*.

eMethods 2. Methods Used to Estimate Private Insurance Contributions and Expenditures

Analyses of persons with private insurance coverage encompassed individuals covered by non-Marketplace as well as Marketplace (Affordable Care Act exchange) private insurance at any time during the past year. We used the policy identification number in MEPS to determine which individuals in a household were covered by each insurance plan. Out-of-pocket premiums (i.e. those paid by the covered individual or someone in their household) for private insurance were estimated from the MEPS. For persons reporting coverage under more than one private insurance plan we summed premiums across all private plans.

Employer contributions to private insurance premiums were estimated using data from the CPS. To calculate the person-level employer contribution for private insurance premiums, we first calculated the total employer contributions for each health insurance unit (usually an individual or family), and then divided this total employer contribution by the number of members in the health insurance unit with private insurance. This yielded a per person estimate of employer contributions. The CPS caps (i.e. top codes) employer contributions, resulting in some under-estimation of such contributions. To address this underestimation, using figures on employer contributions to private insurance from the National Health Expenditure Accounts (NHEA) we first determined the total underestimation of employer-paid premiums in the CPS. We then distributed this figure for the CPS' premium underestimation equally among individuals whose employer contribution was top coded in the CPS.

For exchange insurance, we estimated the Federal subsidy based on MEPS data. Percent subsidies for each individual with private, exchange insurance was estimated based on their poverty level and values from published estimates of exchange subsidy amounts. Each individual's private exchange premium amount was then multiplied by one minus the percent subsidy to estimate the premium paid out-of-pocket after subtracting the subsidy.

All expenditure data were derived from the MEPS, which collects data on expenditures of private insurance on behalf of each respondent, which are verified with providers.

Extreme outliers in MEPS-reported out-of-pocket premium and expenditures for each year of data were identified by log-transforming the data and then applying Tukey's interquartile fence rule to identify outliers. We removed <0.1% of high outlier values each year; there were no extremely low values.

Private insurance (exchange and non-exchange) expenditures measured via MEPS are not expected to sum to insurers premium receipts (employer contributions plus individual contributions) as: 1) premiums include insurance overhead[8] and 2) individuals with high-costs and high service use are known to be underrepresented in MEPS.[9,10] To account for this underestimate, we adjusted our population-wide expenditure estimates to match the population-wide estimates of premiums in the NHEA.

© 2022 Ommerborn MJ et al. *JAMA Network Open*.

eMethods 3. Methods Used to Estimate Contributions to and Expenditures by Medicare

Contributions to the Hospital Insurance Trust Fund (HITF) come primarily from payroll taxes, with smaller amounts coming from income taxes on the Social Security benefits received by higher-income beneficiaries. To calculate payroll contributions to the HITF by immigrants and others, we multiplied each individual's wage and salary earnings from the CPS by 2.9 percent (the Medicare payroll tax rate on most taxpayers). We added revenue from taxes on some Social Security income based on the latest available Congressional Budget Office's (CBO) estimate of those tax rates, which it derived from analyses of the 2005 Current Population Survey and Statistics of Income data published in 2011.[11]

To calculate HITF expenditures for individuals covered by Medicare's fee-for-service program, we summed hospitalization expenditures paid directly by Medicare from the MEPS. Expenditures for those covered under Medicare's managed care (Medicare Advantage) plans were estimated by summing Medicare Advantage plans' total payments to providers and inflating them by the inverse of the average Medicare Advantage medical loss ratio (obtained from a 2009 survey of 41 major Medicare Advantage plans[12]), a standard tool used to adjust payments for administrative and other expenditures by health insurance firms. To tabulate home health care and Medicare Advantage expenditures, we determined the proportion of these expenditures that were financed by the Trust Fund.[13] Trust Fund contribution and expenditure dollar estimates were generated by multiplying each group's share of total contributions (or expenditures) by the Medicare Trustee's estimate of total HITF revenues and outlays for each year of analysis.

Contributions to Medicare Supplemental Medical Insurance Trust Fund (SMITF) come primarily from premiums paid for Part B and Part D insurance and Federal taxes. We estimated premiums for Part B and Part D using data from MEPS. Based on each individual enrollee's annual income and premium rates by income from the Centers for Medicare and Medicaid Services (CMS)[14] we determined how much they would pay annually in Part B or Part D premiums. Federal tax contributions were determined using the CPS. Federal general tax rates from the CBO were applied to individuals based on their household income percentiles. These assigned tax amounts were then divided by family size to distribute tax amounts across the household.

Using the MEPS, expenditures paid by the SMITF were summed to estimate total SMTF expenditures. Expenditures paid by SMITF included: durable medical equipment; home health expenditures not covered under HITF; outpatient care; emergency department care (facility, doctor, and prescription); and inpatient physician fees. As we did for HITF, we generated dollar estimates of SMITF contribution and expenditure by multiplying each group's share of total contributions (or expenditures) by the Medicare Trustee's estimate of total SMITF revenues and outlays for each year of analysis.

HITF and SMITF contributions and expenditures were summed for US-born persons and each immigrant group (citizen immigrants, legal non-citizen immigrant and undocumented immigrants). Total net surplus or deficit was calculated by subtracting the Trust Fund expenditures for each group from the group's Trust Fund contributions.

© 2022 Ommerborn MJ et al. *JAMA Network Open*.

eMethods 4. Methods Used to Estimate Contributions to and Expenditures by Medicaid

We calculated Medicaid contributions separately for residents of each state using data on state taxation rates from the Institute on Taxation and Economic Policy (ITEP); federal tax rate data from the CBO; and data on Medicaid expenditures, state-level Medicaid spending data, and the Federal Medicaid Assistance Percentage (FMAP) from the Kaiser Family Foundation (KFF) (which is derived from Medicaid and CHIP Payment and Access Commission [MACPAC] data).[15,16, 17]

We determined the share of Medicaid spending in each state contributed by the federal government and by the state from the KFF figures on state Medicaid spending and FMAP data. We calculated tax contributions to federal Medicaid spending by applying federal tax rates from the CBO to each individual's household income and family size reported in the CPS. Similarly, we calculated tax contributions to state Medicaid spending by applying state tax rates from ITEP to each individual's reported household income and family size.

Non-nursing home Medicaid expenditures were estimated from MEPS.

© 2022 Ommerborn MJ et al. *JAMA Network Open*.

eMethods 5. Methods Used to Estimate Uncompensated Care Expenditures

The MEPS provides estimates of the cost of uncompensated care delivered at public, but not private facilities. Hence, we estimated total uncompensated care expenditures using American Hospital Association figures[18] and distributed contributions among citizenship/immigration groups in proportion to their share of contributions to the other third-party payers combined. We allocated uncompensated care expenditures among citizenship/immigration groups based on each group's share of the uninsured population, which we calculated from the CPS.

We used CPS population estimates as the denominators to calculate per capita figures. All values were adjusted to 2018 dollars using the Consumer Price Index.[19]

© 2022 Ommerborn MJ et al. *JAMA Network Open*.

eReferences

1. Zallman L, Woolhandler S, Touw S, Himmelstein DU, Finnegan KE. Immigrants Pay More In Private Insurance Premiums Than They Receive In Benefits. Health Aff (Millwood). 2018 Oct;37(10):1663-1668.
2. Zallman L, Wilson FA, Stimpson JP, Bearse A, Arsenault L, Dube B, Himmelstein D, Woolhandler S. Unauthorized Immigrants Prolong the Life of Medicare's Trust Fund. JGIM 2016 Jan;31(1):122-7.
3. Zallman L, Woolhandler S, Himmelstein D, Bor D, MdCormick D. Immigrants contributed an estimated $115.2 billion more to the Medicare trust fund than they took out in 2002-2009. Health Aff (Millwood). 2013;32(6):1153-60.
4. Borjas GJ. The labor supply of undocumented immigrants. Labour Econ. 2017;(46):1-13.
5. United States Census Bureau. Current Population Survey: Methodology. https://www.census.gov/programs-surveys/cps/technical-documentation/methodology.html Accessed March 4, 2022.
6. Pew Research Center. Unauthorized immigrant population trends for states, birth countries and regions. https://www.pewresearch.org/hispanic/interactives/unauthorized-trends/
7. Congressional Budget Office. Federal Subsidies for Health Insurance Coverage for People Under Age 65: 2019 to 2029. May 2019. https://www.cbo.gov/system/files/2019-05/55085-HealthCoverageSubsidies_0.pdf (accessed May 6, 2022).
8. Methodology Report #1: Design and Methods of the Medical Expenditure Panel Survey Household Component. July 1997. Agency for Health Care Policy and Research, Rockville, MD. http://www.meps.ahrq.gov/data_files/publications/mr1/mr1.shtml.
9. Selden TM, Levit KR, Cohen JW, et al. Reconciling medical expenditure estimates from the MEPS and the NHA, 1996. *Health Care Financ Rev*. 2001;23(1):161-178. http://www.hcfa.gov/stats/nhe-oact. Accessed December 31, 2020.
10. Aizcorbe A, Liebman E, Pack S, Cutler DM, Chernew ME, Rosen AB. Measuring health care costs of individuals with employer-sponsored health insurance in the U.S.: A comparison of survey and claims data. *Stat J IAOS*. 2012;28(1-2):43-51. doi:10.3233/SJI-2012-0743
11. Scott C. *Social Security: Calculation and History of Taxing Benefits, CRS Report for Congress*.; 2011. https://greenbook-waysandmeans.house.gov/sites/greenbook.waysandmeans.house.gov/files/2012/documents/RL32552_gb.pdf. Accessed January 29, 2021.
12. *United States House of Representatives Committee on Energy and Commerce Majority Staff, Profits, Marketing and Corporate Expenses in the Medicare Advantage Market.*; 2009.
13. Medicare: Estimated Hospital Insurance Disbursements Calendar Years 1966-2012; Personal Communication with the Deputy Director, Medicare and Medicaid Cost Estimates Group, Office of the Actuary, CMS.
14. The Centers for Medicare and Medicaid Services. Ratebooks and Supporting Documentation. https://www.cms.gov/Medicare/Health-Plans/MedicareAdvtgSpecRateStats/Ratebooks-and-Supporting-Data (accessed June 15, 2022).
15. Kaiser Family Foundation. Total Medicaid Spending. https://www.kff.org/medicaid/state-indicator/total-medicaid-spending/?currentTimeframe=0&sortModel=%7B%22colId%22:%22Location%22,%22sort%22:%22asc%22%7D Accessed May 12, 2022.
16. MACPAC. Exhibit 16. Medicaid spending by state, category, and source of funds. https://www.macpac.gov/publication/medicaid-spending-by-state-category-and-source-of-funds/ Accessed May 12, 2022.
17. The Institute on Taxation and Economic Policy. Who Pays? A Distributional Analysis of the Tax Systems in All Fifty States Sixth Edition. Oct 2018. Accessed June 6, 2022.
18. American Hospital Association. Fact sheet: Uncompensated Hospital Care Costs. https://www.aha.org/fact-sheets/2020-01-06-fact-sheet-uncompensated-hospital-care-cost
19. Bureau of Labor Statistics. Databases, tables, and calculators by subject: CPI Inflation Calculator. https://www.bls.gov/data/inflation_calculator.htm.

© 2022 Ommerborn MJ et al. *JAMA Network Open*.
